# Cis-regulatory sequence variation and association with *Mycoplasma* load in natural populations of the house finch (*Carpodacus mexicanus*)

**DOI:** 10.1002/ece3.484

**Published:** 2013-02-07

**Authors:** Niclas Backström, Daria Shipilina, Mozes P K Blom, Scott V Edwards

**Affiliations:** 1 Department of Organismic and Evolutionary Biology (OEB), Museum of Comparative Zoology (MCZ), Harvard University26 Oxford Street, Cambridge, MA, 02138

**Keywords:** Association mapping, cis-regulatory element, expression, house finch, *Mycoplasma gallisepticum*

## Abstract

Characterization of the genetic basis of fitness traits in natural populations is important for understanding how organisms adapt to the changing environment and to novel events, such as epizootics. However, candidate fitness-influencing loci, such as regulatory regions, are usually unavailable in nonmodel species. Here, we analyze sequence data from targeted resequencing of the cis-regulatory regions of three candidate genes for disease resistance (*CD74*, *HSP90α*, and *LCP1*) in populations of the house finch (*Carpodacus mexicanus*) historically exposed (Alabama) and naïve (Arizona) to *Mycoplasma gallisepticum*. Our study, the first to quantify variation in regulatory regions in wild birds, reveals that the upstream regions of *CD74* and *HSP90α* are GC-rich, with the former exhibiting unusually low sequence variation for this species. We identified two SNPs, located in a GC-rich region immediately upstream of an inferred promoter site in the gene *HSP90α*, that were significantly associated with *Mycoplasma* pathogen load in the two populations. The SNPs are closely linked and situated in potential regulatory sequences: one in a binding site for the transcription factor nuclear NFYα and the other in a dinucleotide microsatellite ((GC)_6_). The genotype associated with pathogen load in the putative NFYα binding site was significantly overrepresented in the Alabama birds. However, we did not see strong effects of selection at this SNP, perhaps because selection has acted on standing genetic variation over an extremely short time in a highly recombining region. Our study is a useful starting point to explore functional relationships between sequence polymorphisms, gene expression, and phenotypic traits, such as pathogen resistance that affect fitness in the wild.

## Introduction

Identification and characterization of the genetic basis of traits affecting fitness differences in nature is key to understanding the forces determining how organisms adapt to the environment (Feder and Mitchell-Olds 2003; Stinchombe and Hoekstra 2008). This search can involve adaptations to varying abundance in different food resources, to changing climate or to exposure to novel pathogen communities. Recent advancements in sequencing and genotyping technologies, allowing for rapid generation of high coverage DNA variant data from large portions of virtually any genome of interest, have made this quest considerably less challenging (Bonneaud et al. 2008; Ekblom and Galindo 2011). However, the successful identification of causal relationships between alleles and the traits that affect the organism's fitness does not solely depend on our ability to generate dense marker maps or genome sequences, but also on access to relevant phenotypic variation. Therefore, forthcoming genotype–phenotype studies in natural populations will likely be centered on taxonomic groups with long-term and/or extensive phenotypic data already on hand (Ellegren and Sheldon 2008). Moreover, many organisms attractive to evolutionary biologists are unsuitable for traditional mapping approaches (Slate 2005) or genome-wide association scans (McCarthy et al. 2008), as pedigree data or adequate population samples might be challenging or impossible to obtain. Until such resources are available, an attractive alternative is to focus on candidate genes known to be associated with the phenotype of interest, in the focal species or in related taxa (Tabor et al. 2002; Piertney and Webster 2010).

The house finch (*Carpodacus mexicanus*) is a sexually dimorphic songbird (Passeriformes: Fringillidae) native to western and southern North America (del Hoyo et al. 2010), and has become an important model for understanding carotenoid-based plumage coloration, sexual selection, and host-pathogen interactions (Hill 1991, 2002; Hill and Farmer 2005). As a consequence of the 19th century pet-trade an introduced population of house finches was founded in the New York City area around 1940, and following geographical expansion and exponential population increase, the house finch became a widespread and common bird in eastern North America, with the number of nesting pairs estimated at several hundred millions (Hill 1993). In 1994, a first case of *Mycoplasma gallisepticum* (MG) infection in house finches was reported from the Washington D.C. area (Ley et al. 1996). The disease, with symptoms including respiratory tract infection and conjunctivitis, resulted in severe population declines throughout eastern populations between 1994 and 1998, after which the first evidence of resistance were observed (Dhondt et al. 1998). Subsequently, MG also spread throughout the eastern US and to native populations in western United States (Cornell Lab of Ornithology 2012). Currently, naïve, MG-unexposed populations can probably only be found in isolated regions of Arizona, Texas, and New Mexico (our unpubl. observations).

MG can affect the immune response in several different ways. In domestic fowl, MG infection may trigger the regulation of chemokines and cytokines to induce an immunological response, but it has also been shown to suppress the immune system at later stages of the response cycle. For example, MG has been shown to inhibit T-cell activity at various points during the adaptive immune response (Razin et al. 1998; Gaunson et al. 2000; Ganapathy and Bradbury 2003; Mohammed et al. 2007). A recently completed series of array-based experiments was designed to understand the potential effects of MG infection on gene expression in the house finch and to identify candidate genes for natural selection during the MG epizootic (Wang et al. 2006; Bonneaud et al. 2011, 2012). By comparing the expression profiles of experimentally infected birds from historically naturally exposed (Alabama) and naïve (Arizona) populations, Bonneaud et al. (2011, 2012) and Wang et al. (2006) identified several candidate genes that may have been involved in the immune response and evolution of resistance to MG in the house finch. This set not only contains obvious disease response-related genes, such as a major histocompatibility complex (MHC) and immunoglobulin genes, but also more generalized genes involved in cellular processes, such as transcription, signaling or stress response pathways (Wang et al. 2006; Bonneaud et al. 2011, 2012). In these studies, a few genes showed intriguing expression differences between Arizona and Alabama birds, as well as between Alabama birds sampled at different time points after the onset of the epizootic (Bonneaud et al. 2011). The genes exhibiting the strongest geographic and temporal expression differences included MHC class II-associated invariant chain (*CD74*), heat-shock protein 90 alpha (*HSP90α*), and lymphocyte cytosolic protein 1 (*LCP1*) (Bonneaud et al. 2011). Both *CD74* and *LCP1* showed decreased expression upon experimental infection with MG, which may indicate modulation of the immune response by naïve birds, or possibly subversion of the immune response by MG. In contrast, *HSP90α*, a gene typically associated with stress (Csermely et al. 1998; Pratt 1998; Feder 1999), was strongly upregulated in experimentally infected birds. The coding regions of all these genes are generally highly conserved, leading us to interrogate the cis-regulatory regions of these genes as potential sources of adaptive variation.

Mutations in cis-regulatory sequences upstream of coding regions, such as transcription factor binding sites and promoters, can play an important role in phenotypic evolution, for example by controlling physiology and development via the regulation of gene expression (Rockman et al. 2004; Hahn 2007; Wray 2007; Chen et al. 2010; Meisel et al. 2012; Wittkopp and Kalay 2012). Cis-regulatory regions are known to have a relatively rapid sequence turnover rate in humans and other eukaryotes (Hahn 2007; Otto et al. 2009) and this has been hypothesized to underlie natural variation in gene expression (Khaitovich et al. 2004, 2005; Wray 2007). However, because of this high rate of sequence turnover, cis-regulatory regions are generally not accessible to those studying nonmodel species, and primers based on one species may not work even in closely related species. We therefore made use of a recently developed genome assembly of the house finch (unpubl. data) to enable a resequencing effort of the upstream regions of the three candidate genes. We hypothesize that, if geographic or temporal changes in gene expression in these genes influence fitness, the signatures of natural selection or associations between cis-regulatory variation and correlates of fitness, such as host pathogen load, might potentially be traced to cis-regulatory sequences located in the upstream regions of the genes (cf. Tung et al. 2009). By comparing samples from naïve and previously exposed populations of house finches, we here characterize patterns of DNA sequence evolution in cis-regulatory regions of a wild bird species and identify putative genetic variants that may be associated with pathogen load and the disease response. Above and beyond any phenotypic associations we observe, our study is noteworthy as the first study (to our knowledge) of sequence variation in cis-regulatory regions of a wild bird species.

## Methods

### Sampling, amplification and sequencing

**Figure 1:**
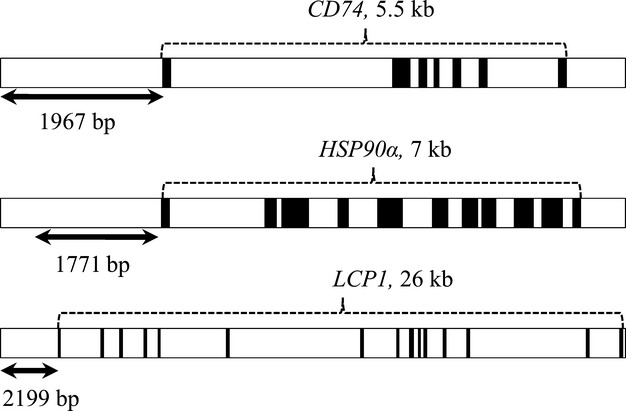
Schematic of the gene structures of *CD74*, *HSP90α* and *LCP1* as annotated in the ensembl genome browser for zebra finch (http://www.ensembl.org/Taeniopygia_guttata). Exons are indicated with black boxes and introns and untranslated regions with white boxes. The length (kilobases, kb) of the transcribed region is given after the gene name and the region sequenced (arrow) and its length in base-pairs (bp) is indicated under each gene. The bracketed interval indicates the coding portion plus introns.

We used DNA from house finches sampled in 2007 from (1) a population that had previously been exposed to MG (Alabama, *n* = 24); and (2) from a population naïve to MG in nature (Arizona, *n* = 24) (Bonneaud et al. 2011). Additionally, this group contained a subset of birds with data on pathogen load with which we might associate promoter sequence variation (Bonneaud et al. 2011; see below). The mRNA sequences for *CD74*, *HSP90α*, and *LCP1* used for microarray analysis (Bonneaud et al. 2011) were used in BLAST searches to identify the relevant scaffolds in the draft house finch genome assembly (our unpubl. data; alignments of the focal regions have been submitted to the Dryad database (http://datadryad.org/) under accession doi:10.5061/dryad.5p91k). The identified scaffold sequences were used to design primers for amplification of upstream regions of each of the three genes. We aimed to sequence at least 1.5 kb of the region immediately upstream (5′) the first codon position. To confidently cover the boundary between the first exon and the upstream region, at least one primer was designed to match an internal segment of exon 1. For *HSP90α* the intron–exon structure adjacent to exon 1 was not well defined and we therefore decided to sequence parts of intron 1 too. Gene structures and the regions sequenced for each gene are presented in Fig. 1 and all primer sequences are presented in Table S1. Amplifications used approximately 50–100 ng template DNA in a 25 μL reaction together with 5 μL LongAmp (New England Biolabs, Ipswich, MA, USA) buffer (including 2 μM MgSO_4_), 0.75 μL dNTP mix (10 μM), 14.75 μL double-deionised water, 1 μL each of forward and reverse primer (10 μM), and 1 μL LongAmp DNA Polymerase (New England Biolabs, Ipswich, MA, USA) on a Mastercycler thermal cycler (Eppendorff AG, Hamburg, Germany). For amplification of GC-rich regions, we added either DMSO or Betaine to the reaction mix. The general temperature profile was an initial 30 sec denaturation step at 94°C followed by 21 cycles, including temperatures 94°C (20 sec), 63–53°C (45 sec, decreasing 0.5°C per cycle), and 65°C (60 sec) and 20 cycles with identical settings but keeping the annealing temperature constant at 53°C and finally, a 10-min elongation step at 65°C. Some primer combinations required optimization and PCR settings for specific amplicons are available from the authors upon request. PCR products were run on a 2% agarose gel stained with SYBR safe (Invitrogen, Life Technologies Corp., Grand Island, NY, USA) and visually inspected for specificity. Amplification products were purified using ExoSAP-IT following manufacturer's recommendations (USB Corp., Cleveland, Ohio, USA). Sequencing reactions were performed in 10 μL volumes, including forward or reverse primer and following the recommendations for the BigDye 3.1 chemistry (Applied Biosystems, Life Technologies Corp., Carlsbad, CA, USA) and the BDX64 buffer solution (MCLAB, South San Francisco, CA, USA). Sequencing reactions were purified using sephadex gel filtration in 96-well microtiter plates (GE Healthcare, Waukesha, WI, USA) and sequencing was carried out on ABI3730xl and ABI3130xl sequencers (Applied Biosystems, Life Technologies Corp., Carlsbad, CA, USA).

### Population genetic analysis

Sequences were manually edited in Sequencher (Gene Codes Corp., Ann Arbor, MI, USA) and aligned using Clustal W (Thompson et al. 1994) as implemented in Mega 4 (Tamura et al. 2007) or in Geneious 4.9 (Biomatters Ltd., Auckland, New Zealand). Contigs were used in BLAST (Altschul et al. 1990) searches at the ensembl genome browser web portal using the zebra finch genome sequence (http://www.ensembl.org/Taeniopygia_guttata/) as reference to verify orthology to the target gene. Following inference of haplotypes for each individual in DAMBE (Xia and Xie 2001), we calculated basic population genetic summary statistics (π, Tajima's *D* and *F*_*ST*_) for noncoding regions in DnaSP 5 (Librado and Rozas 2009), excluding sites with missing data only in pair-wise comparisons; all birds could not be sequenced for the entire stretch of each gene, as multiple indels were segregating in the sample set. Confidence limits for Tajima's *D* were estimated by 1000 random permutations assuming a moderate level of recombination (the population recombination rate, ρ = 4N_e_ *r* = 10 per locus). We applied the Bayesian modeling approach implemented in BAYESFST (Beaumont and Balding 2004) to test if any SNP was more differentiated than expected. This test was run for SNPs from single genes separately and for all SNPs combined. Haploview 4.2 (Barrett et al. 2005) was used to infer linkage disequilibrium (LD, *r*^2^) between all SNPs with minor allele frequency >10% in each population.

### Tests of genotype-pathogen load associations

Previously available pathogen load data for a subset of the sequenced individuals (*n* = 9 and 8 for Alabama (exposed) and Arizona (naïve), respectively; Bonneaud et al. 2011) was used to investigate potential associations between the pathogen load (ratio of MG to host cells 2 weeks postexperimental infection) and SNP genotypes. We selected only SNPs with high enough minor allele frequency (MAF) to potentially detect a significant association after Bonferroni correction for multiple testing. The Tukey–Kramer pair-wise comparison of means as implemented in the R package Multcomp (a statistical framework for testing general linear models; Hothorn et al. 2008) was used to test for potential association between genotype and pathogen load. For each SNP included in the association analyses, we calculated expected genotype frequencies based on allele frequency data and tested for Hardy–Weinberg equilibrium using the RGenetics project package (http://rgenetics.org). To link any possible SNP associations to regulatory features of these promoters, we scanned the sequenced regions for occurrence of putative transcription factor binding sites using the search algorithm implemented in the core vertebrate database Jaspar (Bryne et al. 2008), using all known transcription factors from chicken (*Gallus gallus*) and a 90% similarity threshold.

## Results

### Cis-regulatory sequence variation

We resequenced approximately 1.5–2 kb of the upstream region of *CD74*, *HSP90α* and *LCP1,* three candidate genes for MG resistance in the house finch. GC base composition varied considerably among genes (mean GC = 54.7, 57.2 and 43.3% for *CD74*, *HSP90α* and *LCP1*, respectively; Table 1) and between regions within genes. The number of segregating sites and the average genetic diversity (within populations and overall) was highly variable among regions (Table 1). The lowest number of polymorphic sites (*n* = 5) and number of private and shared SNPs was observed for *CD74* (total 1967 bp). In contrast, in the upstream region of *HSP90α* (1771 bp), we observed 72 SNPs, with a moderate number of shared and private SNPs. The *LCP1* upstream region (2199 bp) exhibited a level of variation similar to the *HSP90α* upstream region, with 88 SNPs. Overall genetic diversity was lowest in *CD74* (π = 5.1e^−5^), much lower than the diversity in *LCP1* (π = 1.8e^−3^) and *HSP90α* (π = 2.0e^−3^). The biggest difference in genetic diversity among populations was observed in *HSP90α*, with twice as much diversity in AZ as compared to AL (Table 1). Tajima's *D* values were overall positive in AL, but overall negative in AZ (Table 1).

**Table 1:** Summary statistics for the three genes included in the study

	Length (bp)	GC %	S_tot_	S_share_	S_AL_	S_AZ_	π_tot_	π_AL_	π_AZ_	*D*_AL_	*D*_AZ_	*F*_*ST*_	Indels
*CD74*	1967	54.7	5	2	0	3	5.1e^−5^	6.9e^−5^	3.7e^−5^	0.74	−0.55	0.000	2 (1,2)
*HSP90α*	1771	57.2	72	30	14	28	2.0e^−3^	1.3e^−3^	2.5e^−3^	0.56	−0.74	0.030	4 (1,1,3,16)
*LCP1*	2199	43.3	88	66	11	10	1.8e^−3^	1.7e^−3^	1.8e^−3^	0.18	−0.07	0.014	4 (1,1,1,1)

GC% = percentage of guanine and cytosine bases in the region. S = number of SNPs in total (tot), shared (share) and for each population (AL and AZ, respectively). *D* = Tajima's *D*, *F*_*ST*_ = coefficient of differentiation (Wright's F-statistic) between populations, Indels = the number of insertion/deletion polymorphisms with the length in base-pairs of each respective indel given within parentheses.

**Figure 2:**
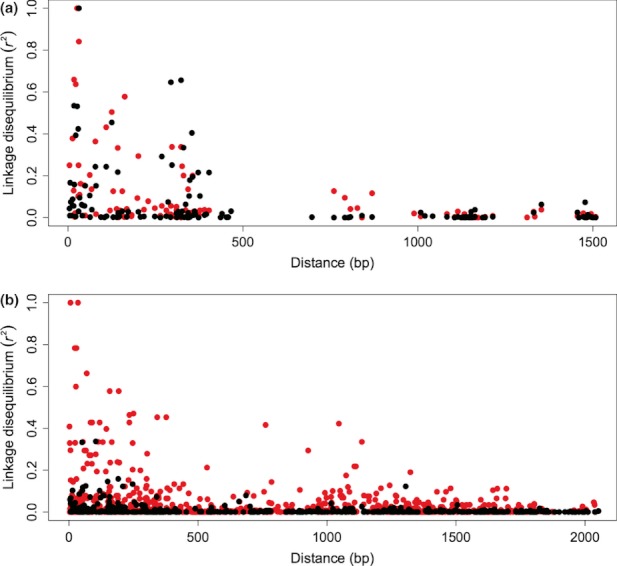
Scatterplot of the pair-wise linkage disequilibrium (*y*-axis) and physical distance (*x*-axis) between SNPs with minor allele frequency > 10% within the genes *HSP90α* (a) and *LCP1* (b) for the Arizona (black) and Alabama (red) populations. *CD74* did only contain a single pair of SNPs with MAF > 10% (distance = 1562 bp apart, *r*^2^ = 0.0060) and is therefore excluded from the figure.

None of the three genes showed significant differentiation between AL and AZ populations, as measured by *F*_*ST*_ (Table 1). The outlier analysis (BAYESFST) did not detect a signal of divergent selection, neither when SNPs within genes were analyzed separately nor when all SNPs were combined (Figure S1). We estimated LD between pairs of SNPs with MAF >10% and plotted that against physical distance between markers and found that LD decays extremely rapidly in both *HSP90α* and *LCP1* in both populations (Fig. 2). *CD74* only contained a single pair of SNPs with MAF > 10% (distance = 1562 bp apart, *r*^2^ = 0.0060 and 0.0070 for Alabama and Arizona, respectively).

**Figure 3:**
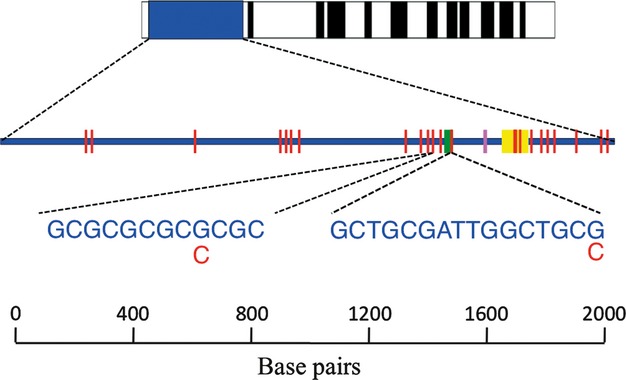
Illustration of the sequenced region of *HSP90α*. The red bars indicates the SNPs included in the association test, the pink bar is the likely promoter region, the yellow block is the first exon and the green block denotes the putative nuclear factor NFYα binding site. The position of the six unit dinucleotide microsatellite is also shown.

When using the sequence from previously described transcription factors identified in the domestic fowl as a reference and listed in the Jaspar database, we identified between four and 20 putative transcription factor binding sites in the upstream region of the sequenced genes (Table 2). In total we identified 38 potential binding sites, the majority (*n* = 27) of which corresponded to the short and somewhat redundant zinc-coordinating ββα-zinc finger factor ZEB1 (Table 2). In addition, across all three promoters, we identified 10 regions that matched binding sites for the leucine zipper NFE2L1 and a single binding site in the upstream region of *HSP90α* for the nuclear factor NFYα (Table 2, Fig. 3).

**Table 2:** The number (#) of putative transcription factor binding sites identified in the upstream region for each of the three genes and in total and the corresponding amount of sequence data covered by these sites (bp)

Gene	# ZEB1 – zinc coordinating	ZEB1 (bp)	# NFE2L1 – leucine zipper	NFE2L1 (bp)	# NFYα – nuclear factor	NFYα (bp)	Total #	Total (bp)
*CD74*	2	12	2	12	–		4	24
*HSP90α*	17	102	2	12	1	16	20	130
*LCP1*	8	48	6	36	–		14	84
Total	27	162	10	60	1	16	38	238

Sequence surveys of cis-regulatory regions in natural populations are sparse, but the few that are available report that potential functional sites harbor similar or slightly higher levels of variation as compared with the surrounding regions (Balhoff and Wray 2005; Brown and Feder 2005; Garfield et al. 2012). To investigate this in the house finch, we quantified SNP variation (nucleotide diversity, π) within and outside transcription factor binding sites and found that levels of variation in putative transcription factor binding sites varied extensively among genes (Table 3). For *CD74*, we did not have any sequence polymorphisms within the putative binding sites, whereas the rates were higher within binding sites in *HSP90α* and *LCP1*. However, given the limited data for binding sites, the variance estimates for π are high and we cannot say that these are significantly different from rates occurring outside binding sites (Table 3). Indel variation in the three upstream regions was negligible, with only 2–4 short indels occurring throughout the regions (Table 1). None of these showed association with pathogen load and only one overlapped a potential transcription factor binding site (a 16-bp indel partly overlapping a putative ZEB1 binding site in *HSP90α*).

**Table 3:** The overall nucleotide diversity (π) within putative transcription factor binding site classes; in the total region covered by transcription factor binding sites; and in regions outside binding sites in the upstream region of each of the three genes

Gene	π_ZEB1_	π_NFE2L1_	π_NFYα_	π_TOTAL_	π_OUTSIDE_
*CD74*	0 (12)	0 (12)	NA (0)	0 (24)	0.000052 ± 0.000051 (1943)
*HSP90α*	0.0017 ± 0.033 (102)	0 (12)	0.031 ± 0.025 (16)	0.0051 ± 0.029 (130)	0.0019 ± 0.0014 (1641)
*LCP1*	0.0073 ± 0.047 (48)	0 (36)	NA (0)	0.0042 ± 0.041 (84)	0.0017 ± 0.0013 (2115)

The total number of base-pairs covered by the motif or class is given within parentheses.

### Association of SNP and pathogen load variation

**Figure 4:**
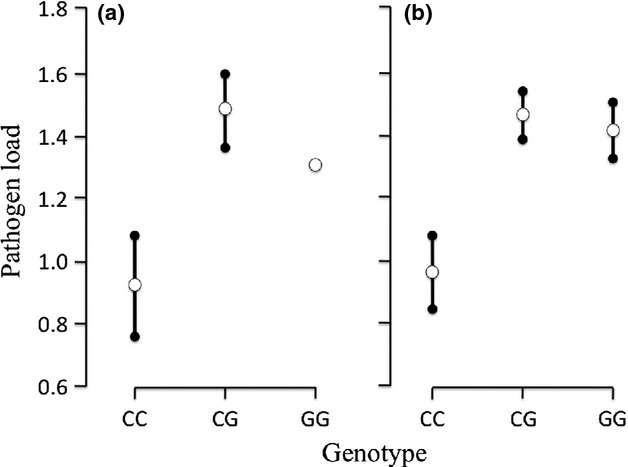
The pathogen load (*y*-axis) as measured by the ratio of pathogen cell to host cell number for groups of individuals with different genotypes for the two positions SNP1558 (A, *n* = 17) and SNP1620 (B, *n* = 17). In both cases did the C/C genotype birds show significantly lower pathogen load (Tukey-Kramer test, corrected *P*-value < 0.05) than birds with the C/G or G/G genotype.

**Figure 5:**
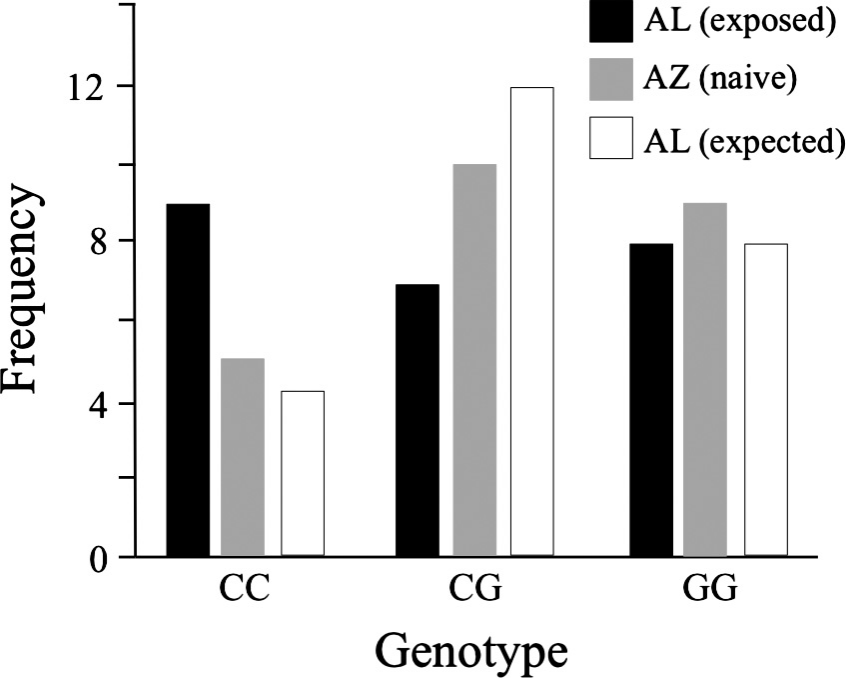
Histogram showing the observed frequency of the different genotypes in the historically exposed AL population (black), the naïve AZ population (gray), and the expected genotype frequencies given the allele frequencies in the sample (white). The AZ population conforms to HWE but the AL population deviates significantly from HWE, predominantly as a result of excess of individuals with the C/C genotype (HWE test, *P*-value < 0.05).

**Figure 6:**
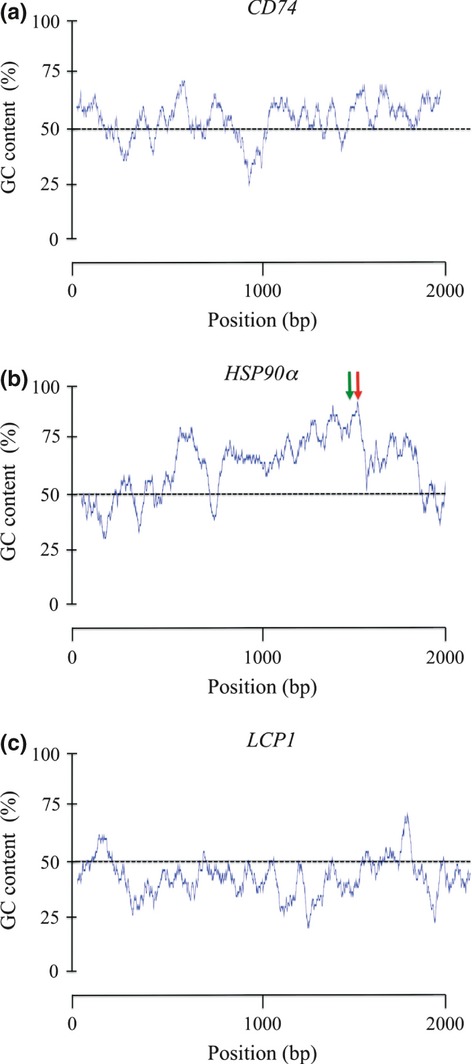
The GC content (50 bp sliding window) in the upstream region of *CD74* (a), *HSP90α* (b) and *LCP1* (c). Arrows in (b) indicate the position of the NFYα transcription factor binding site in the *HSP90α* promoter (red) and the dinucleotide microsatellite ((CG)_6_, green), both of which contain SNPs associated with pathogen load.

We used previously available pathogen load data for a subset (*n* = 17) of the resequenced individuals to investigate a potential association between genotype and *Mycoplasma* load. After omitting SNPs that had too low MAF to detect a signal (see methods) there were 0, 24, and 22 SNPs available for analysis in *CD74*, *LCP1*, and *HSP90α*, respectively. The 22 SNPs in the *HSP90α* region included two SNPs in exon 1 and five SNPs in intron 1. We found no significant association between genotype and host *Mycoplasma* load for SNPs in *LCP1*, but in *HSP90α,* two SNPs (henceforth SNP1558 and SNP1620), both C/G polymorphisms and located within 62 bp of each other, showed significantly lower pathogen load for the C/C genotype (Tukey–Kramer test, corrected *P*-value < 0.05) as compared to other genotypes (C/G or G/G, Fig. 4). For SNP1620, there was a significantly higher number of C/C homozygotes (the genotype associated with lower pathogen load) than expected from random association of alleles (Hardy–Weinberg test, *P*-value < 0.05) in the AL birds, but not in the AZ sample (*P*-value > 0.05). By contrast, there was no deviation from HW expectations in either population for SNP1558 (Fig. 5). Both SNP1558 and SNP1620 are located in a GC-rich region (Fig. 6) around 150 bases upstream of a putative promoter sequence (TATAAAT) 20 bases upstream of the start of exon 1 (Fig. 3). SNP1558 is located within a six unit dinucleotide ((CG)_6_) repeat sequence, and, intriguingly, SNP1620 occurs in the 3′ end of the only identified transcription factor binding site for NFYα (Figure 3). We regard these as two independent associations of SNPs with pathogen load, given that linkage disequilibrium between the two loci is low (*r*^2^ = 0.45 and 0.32 and |D′| = 0.87 and 0.84 in the Alabama and Arizona populations, respectively; Figure S2).

## Discussion

We performed targeted resequencing of approximately 2 kb of the upstream region of three genes, *CD74*, *LCP1*, and *HSP90α*, previously shown to exhibit significant expression changes in experimentally infected house finches from naturally naïve (AZ) and previously exposed (AL) populations. Two SNPs, both located close to each other (62 bp apart) in a GC-rich region ∼150 bases upstream of an inferred promoter site in *HSP90α*, a gene that plays an important role in the immune response (Feder 1999; Srivastava 2002; Wallin et al. 2002; Tsan and Gao 2009), showed significant association with pathogen load. Interestingly, one of the SNPs (SNP1620) was located in the 3′ end of a putative binding site for the transcription factor NFYα, a transcription-regulating metalloprotein, which binding is highly dependent on the sequence within the binding region (Dorn et al. 1987; Maity and de Crombrugghe 1998). NFYα is known to regulate transcription of a large set of genes and has a role in immune system regulation by regulating expression of the major histocompatibility complex (MHC) class II genes (Benoist and Mathis 1990; Li et al. 1991; Jabrane-Ferrat et al. 2002; Hunt et al. 2005). The other SNP showing significant association with pathogen load (SNP1558) was located within a six unit dinucleotide microsatellite (CG_6_) only 62 bases upstream of SNP1620. Earlier efforts have shown that tandem repeat structures often coincide with regulatory motifs and that variation in number of repeat units can affect transcription rate and the resulting phenotype (Gemayel et al. 2010). Previous studies also indicate a positive correlation between GC content and the number of regulatory sequences in a genomic region (Hapgood et al. 2001; Bachmann et al. 2003) and, interestingly, both of the focal SNPs were located in a region that harbors particularly high GC content (76%, Fig. 6), by far the highest in any of the investigated regions. These results are all consistent with previous evidence implicating *HSP90α* as a candidate gene for the evolution of resistance to *Mycoplasma* in the house finch (Wang et al. 2006; Bonneaud et al. 2011, 2012).

An alternative explanation for the finding of two SNPs associated with pathogen load is that one of the SNPs has been under selection and that hitchhiking has caused the significant association at the other site. However, linkage disequilibrium between the two loci is low suggesting that hitchhiking has probably been a weak force in this region. Additionally, population structure is known to be problematic for association studies, often causing false positives when structure covaries with the phenotype of interest (Hirschhorn and Daly 2005; Roeder and Luca 2009; Price et al. 2010). However, despite their wide geographic spread, we found little or no population structure in our sample of AZ and AL birds, consistent with a mild population structure in the house finch (Wang et al. 2003; Hawley et al. 2006). The distribution of pathogen load scores in our sample differed significantly between AZ and AL birds (Wilcoxon's test, W = 11, *P*-value = 0.019), potentially confounding our detected associations. However, when applying a linear model and treating the pathogen load as response variable and both genotype and population as explanatory variables, genotype was still significantly associated with pathogen load (df = 2, *F* = 9.05, *P*-value = 0.0035), whereas population was not (df = 1, *F* = 0.89, *P*-value = 0.36). Finally, the *F*_*ST*_-estimate for the two focal SNPs was among the lowest for all SNPs detected in the study (Figure S1), a situation that would mitigate against false associations.

At both SNPs the C alleles were present in the naïve AZ population and therfore represent standing variants already present in house finches before the encounter with MG. Under the likely assumption that reduced disease susceptibility should have been selected for in the eastern US (AL) population, we assessed if the genotypes associated with lower pathogen load (C/C) were segregating at expected frequencies given the allele frequencies of each variant (Hardy–Weinberg Equilibrium, HWE) at the two loci. SNP1558 did not show any deviation from HWE, but at SNP1620, the C/C genotype was significantly overrepresented in the historically exposed AL population, but not in the naïve AZ population. Hence, it is possible that exposure to MG over the ∼13 years between the MG outbreak in 1994 and our sampling in 2007, has shifted genotype frequencies in the AL population, favoring individuals with a genotype associated with lowered pathogen load (C/C). We also observe a lower than expected frequency of the C/G genotype in the AL population, as is expected if the C/C genotype has been selected for and very recently (i.e., before complete random mixing of alleles in the population again) increased the frequency of the C allele in the population.

The first case of MG was observed in eastern United States in 1994, 13 years prior to the sampling of individuals used in this study. Given the severe effects of the MG epizootic, directional selection on alleles with an effect on disease susceptibility has probably been extraordinarily strong in exposed populations. In the upstream region of *HSP90α*, we observed slightly reduced nucleotide diversity in AL as compared with AZ and the Tajima's *D* was positive in AL, indicating a lack of low frequency variants. However, the Tajima's *D* did not deviate significantly from 0 and was not different from estimates from the other two genes. The decay of linkage disequilibrium in *HSP90α* was as rapid in the Alabama population as in the Arizona population, although the LD was slightly higher between the two alleles associated with lower pathogen load in the Alabama birds (Figure S2a). Hence, comparable to what has recently been found in MHC related genes (Hawley and Fleischer 2012), it is possible that a combination of a high local rate of recombination and selection acting on recessive standing genetic variants for a too short period of time to severely reduce diversity, drive detectable local differentiation, inflate local linkage disequilibrium or cause significantly deviating allele frequencies at linked sites.

Our sampling and use of sequence-based markers (Backström et al. 2008; Brito and Edwards 2009) allowed us to calculate population statistics, such as π and Tajima's *D*, something that previous phylogeographic studies in the house finch have been unable to do (Wang et al. 2003; Hawley et al. 2006, 2008). The genetic diversity varied significantly between genes, *CD74* having much lower diversity than *LCP1* and *HSP90α*. Genetic diversity at *LCP1* and *HSP90α* fell just below ranges previously observed for an anonymous locus (*ALHF1*, π ≍ 5e^−3^) in the house finch (Hess et al. 2007), whereas the estimate for *CD74* was two orders of magnitude lower and well below the level of diversity generally observed in putatively unconstrained regions for birds (ICPMC 2004; Backström et al. 2008; Balakrishnan and Edwards 2009; Balakrishnan et al. 2010; Warren et al. 2010), suggesting strong evolutionary constraint. GC content varied considerably between genes and between different regions within the upstream sequence analyzed. For *CD74* and *HSP90α*, the GC content was substantially higher than the genome-wide GC content observed in birds generally (ICGSC 2004; Dalloul et al. 2010; Warren et al. 2010) and similar to transcribed portions of the house finch genome (56%; Backström et al. 2012). Tajima's *D* was found to be positive for all genes in the AL population, but overall negative in the AZ population, in agreement with expectations after a recent founder event that could have been caused by the *Mycoplasma* epizootic or by the human-induced bottleneck on eastern US populations, a point on which previous studies have varied (Wang et al. 2003; Hawley et al. 2006, 2008). However, the overall nucleotide diversity is similar between the two populations, indicating that neither the putative founder event nor the epizootic resulted in a reduction in diversity at these loci (cf. Wang et al. 2003; Hawley et al. 2006, 2008; Hess et al. 2007).

## Conclusions

Our study is a first attempt to characterize DNA sequence patterns in potential regulatory regions in a wild bird species. From a pool of 165 SNPs, we identified two closely linked SNPs that were significantly associated with the phenotypic response to an infectious disease in natural populations of the house finch. Both polymorphisms were located in putative regulatory sequences in a GC-rich region just upstream of a likely promoter site in *HSP90α*, a gene that previously has been implicated in stress response in model organisms. One of the regulatory sequences was the only detected binding site for a transcription factor, nuclear factor NFYα, previously associated with transcriptional regulation of the MHC class II immune response gene cluster, as well as other relevant genes. Furthermore, the genotype associated with lower pathogen load was significantly overrepresented in Alabama birds, which have been historically exposed to MG. Taken together, all these observations suggest a functional role for at least one of these polymorphisms. Although we fail to identify strong signals of selection acting on the locus and our observations are only correlations, this work presents an important starting point, potentially leading to experiments involving artificial selection or synthetic promoter regions to explore functional links between DNA sequence variation, gene expression, and fitness.

## Supporting Information

**Figure S1.** Individual *F_ST_* estimates (*y*-axis) and their corresponding *P*-values (*x*-axis scaled as 2*abs(0.975–0.5)) for all SNPs identified in the three genes when analyzed using the BAYESFST method (Beaumont and Balding 2004). The vertical dashed red line indicates the threshold for significance (*P* = 0.05). Values to the right of the vertical bar would be significant at the 0.05 level. The SNPs associated with pathogen load are indicated in green (SNP1558) and red (SNP1620).

**Figure S2.** Plots of pair-wise linkage disequilibrium between SNPs with minor allele frequenc *y* >10% located within each of the genes *HSP90*α(a, b) and *LCP1* (c, d) for the Alabama population (a, c) and the Arizona population (b, d), respectively. The horizontal bar indicates the sequenced region and the vertical lines show positions of SNPs with minor allele frequency >10% along the stretch. SNsP numbers are given below the horizontal bar and numbers within diamonds denote the *r*^2^-values. The level of LD is also indicated by the shading of the diamond representing a SNP pair, the darker the shading the higher the LD (white indicates *r*^2^-values = 0 and black indicates *r*^2^-values = 1). *CD74* did only contain a single pair of SNPs with MAF > 10% and is therefore omitted from the figure.

**Figure S3.** Schematic of the relative position of primers for each of the three genes. Primer sequences are given in Table S1. Forward primers are in red and reverse primers in blue. Black horizontal bars indicate the sequence region analyzed for each gene.

**Table S1.** Primer combinations used for amplification of the upstream region of the three candidate genes. As a result of varying GC content and several polymorphic length variants more than one primer combination had to be used to cover the region. The specific PCR settings for each amplicon are available upon request. For approximate locations see Figure S3.
