# Supplementary material for: Cis-regulatory sequence variation and association with Mycoplasma load in natural populations of the house finch (Carpodacus mexicanus)
Source: Ecol Evol. 2013 Feb 7;3(3):655–66. doi: 10.1002/ece3.484 (PMC3605853; doi:10.1002/ece3.484)
Supplement: Supplementary file 1 [file ece30003-0655-SD1.pdf]

Supplementary Information

Supplementary Figure 1

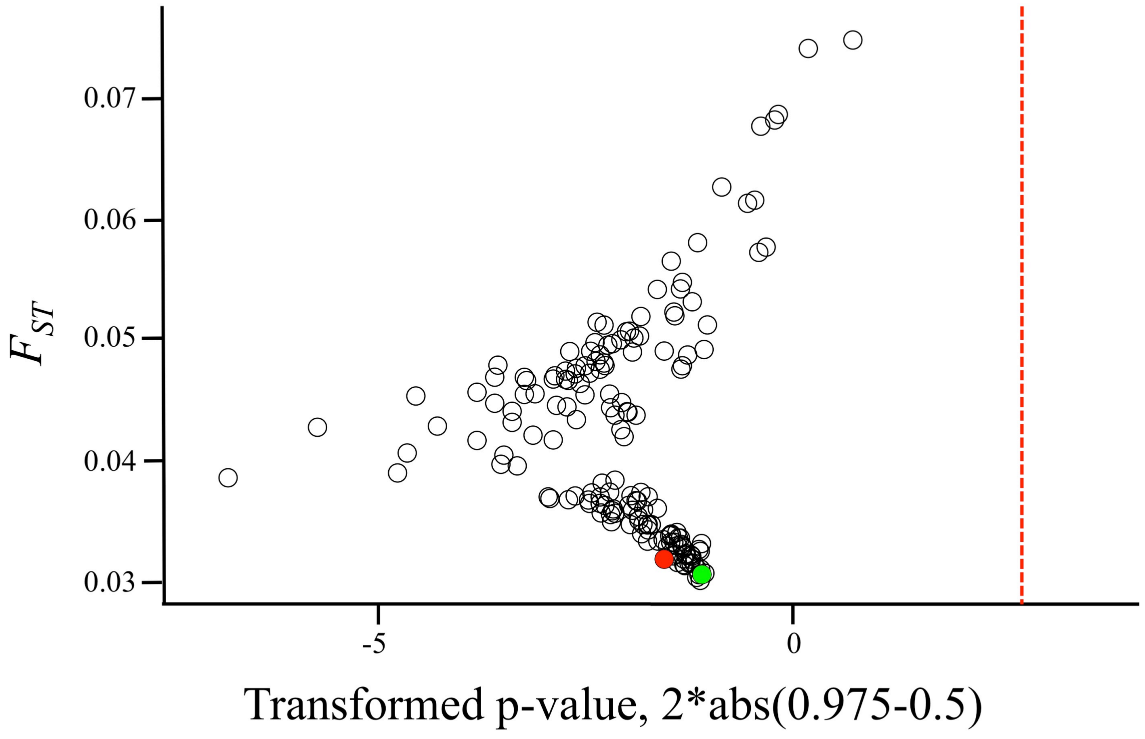

Supplementary Figure 1

Individual F_{ST} estimates (y-axis) and their corresponding p-values (x-axis scaled as 2*abs(0.975-0.5)) for all SNPs identified in the three genes when analysed with the BAYESFST method (Beaumont and Balding 2004). The vertical dashed red line indicates the threshold for significance (p = 0.05). Values to the right of the vertical bar would be significant at the 0.05 level. The SNPs associated with pathogen load are indicated in green (SNP1558) and red (SNP1620).

Supplementary Figure 2

a (*HSP90α* Alabama)

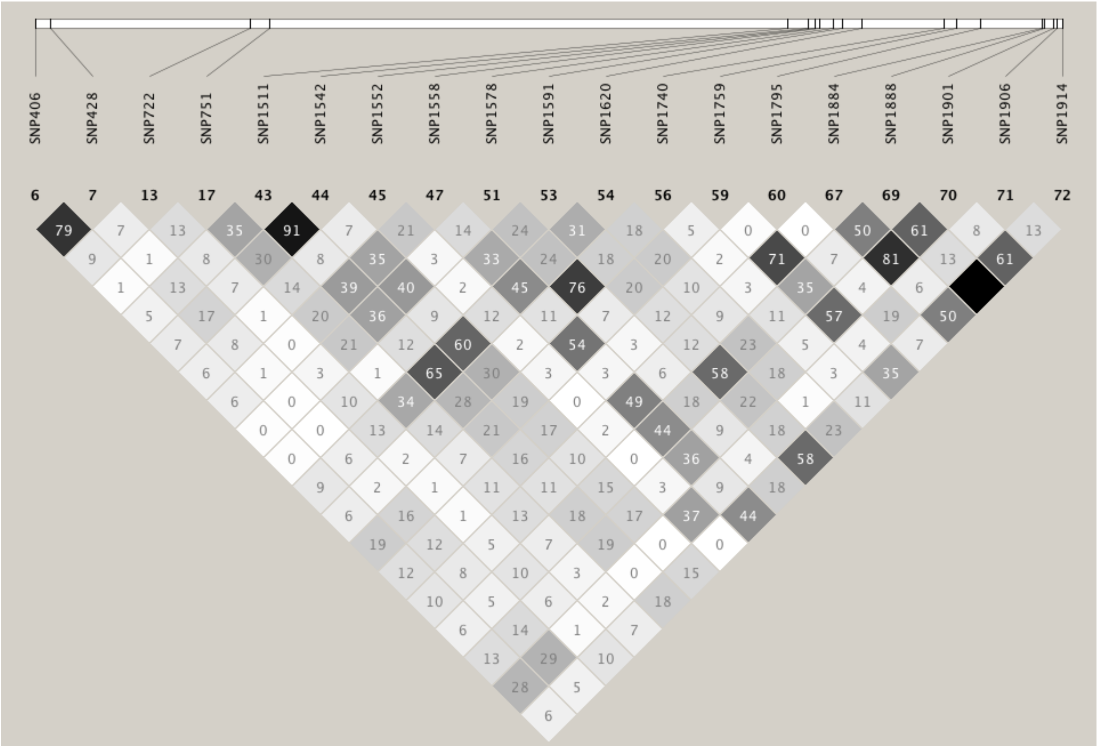

b (*HSP90α* Arizona)

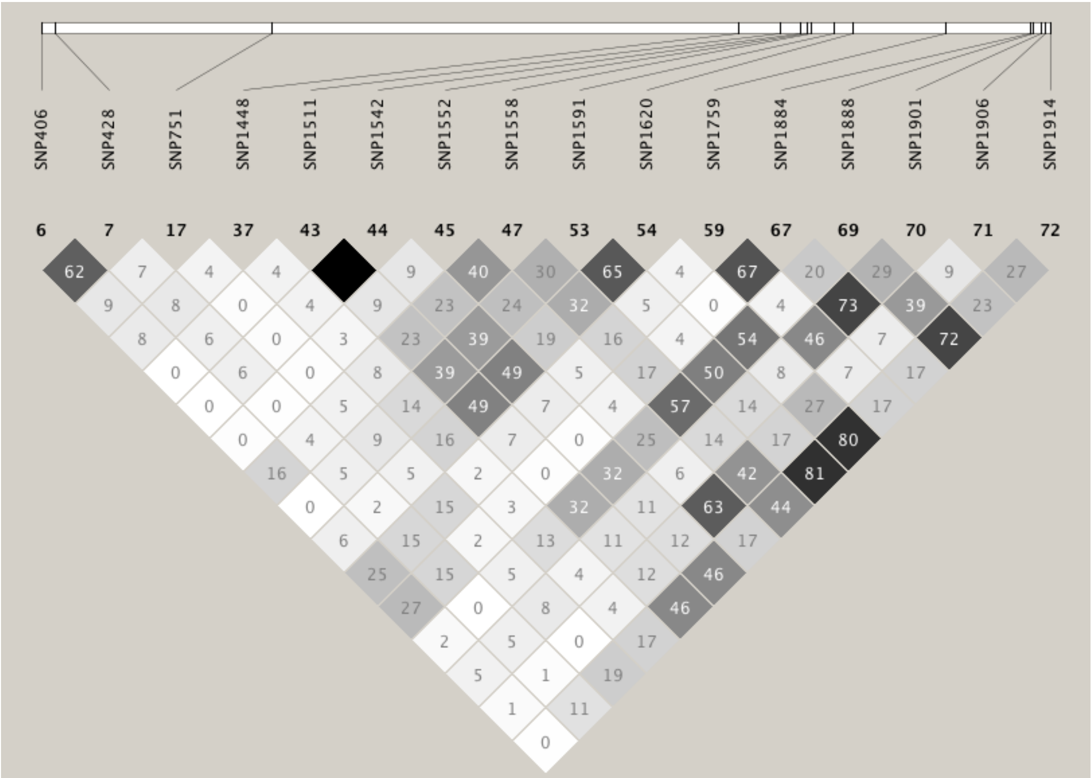

Supplementary Figure 2 continued

c (*LCP1* Alabama)

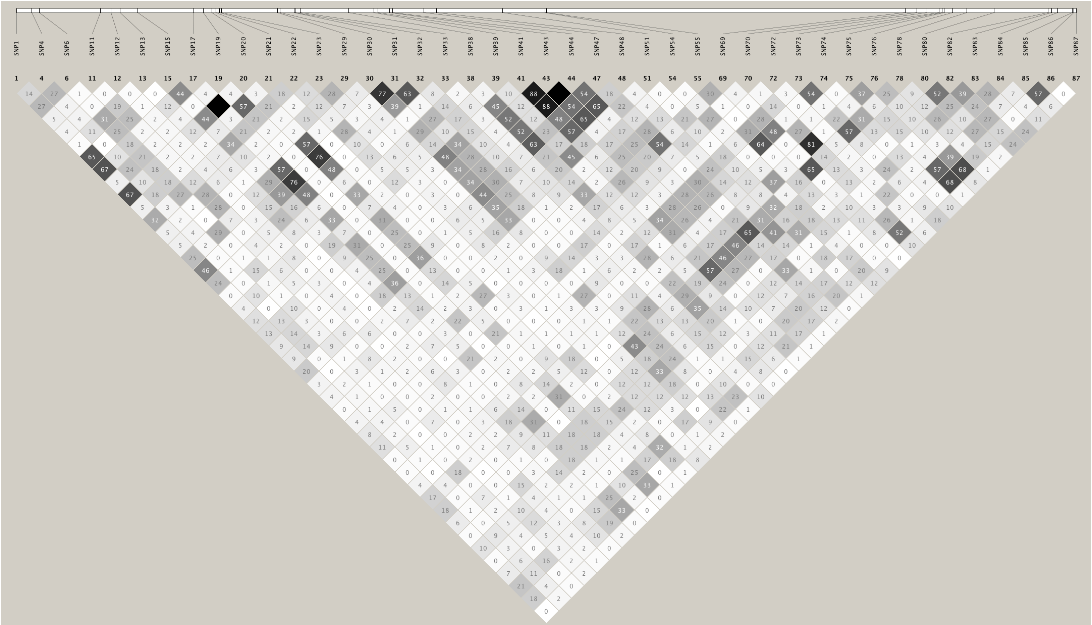

d (*LCP1* Arizona)

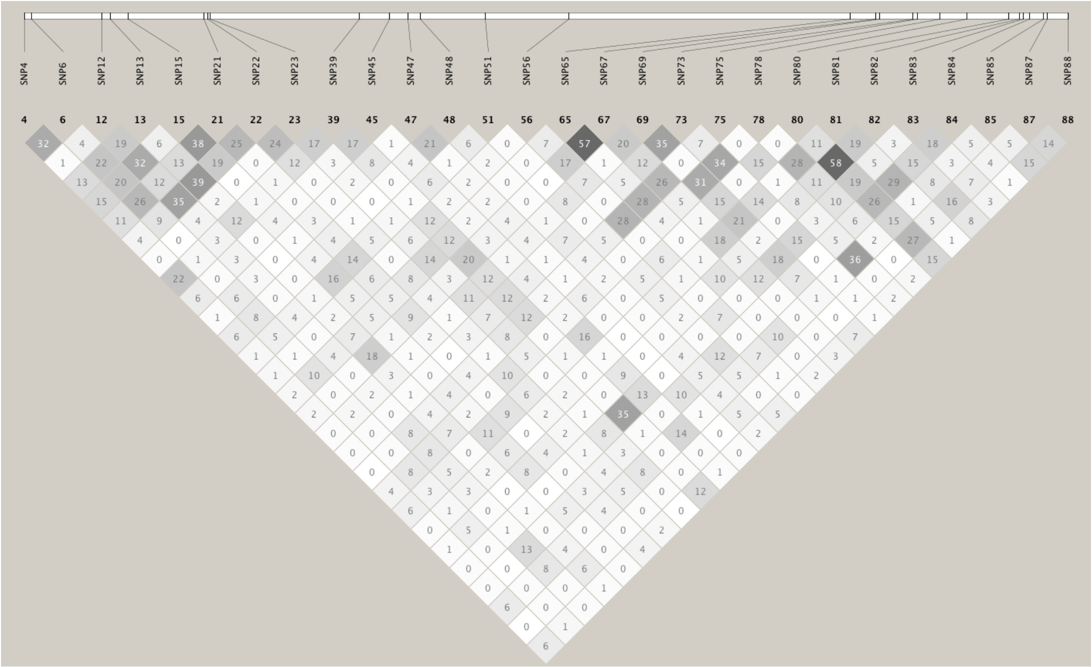

Supplementary Figure 2 continued

Supplementary Figure 2

Plots of pair-wise linkage disequilibrium between SNPs with minor allele frequency > 10% located within each of the genes *HSP90*α (a, b) and *LCP1* (c, d) for the Alabama population (a, c) and the Arizona population (b, d), respectively. The horizontal bar indicates the sequenced region and the vertical lines show positions of SNPs with minor allele frequency > 10% along the stretch. SNP numbers are given below the horizontal bar and numbers within diamonds denote the r^2-values. The level of LD is also indicated by the shading of the diamond representing a SNP pair, the darker the shading the higher the LD (white indicates r^2-values = 0 and black indicates r^2-values = 1). *CD74* did only contain a single pair of SNPs with MAF > 10 % and is therefore omitted from the figure.

Supplementary Figure 3

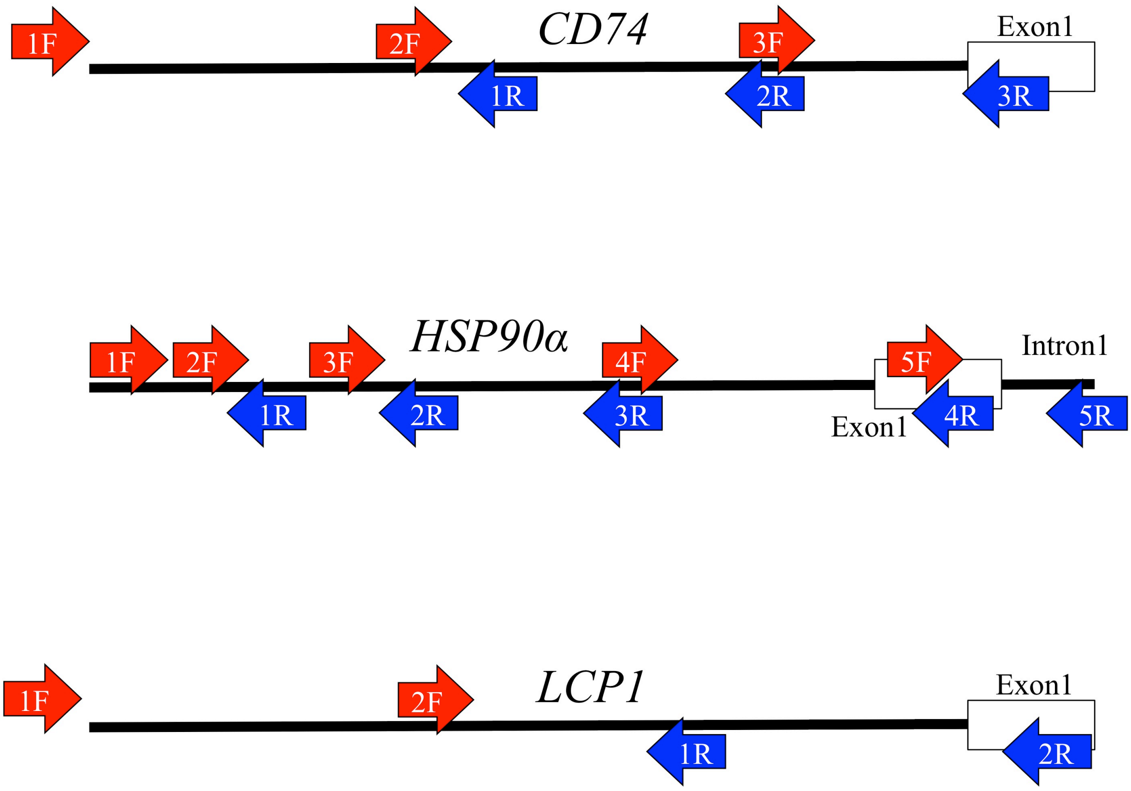

Supplementary Figure 3

Schematic of the relative position of primers for each of the three genes. Primer sequences are given in Supplementary Table 1. Forward primers are in red and reverse primers in blue. Black horizontal bars indicate the sequence region analyzed for each gene.

Supplementary Table 1.

Primer combinations used for amplification of the upstream region of the three candidate genes. As a result of varying GC content and several polymorphic length variants more than one primer combination had to be used to cover the region. The specific PCR settings for each amplicon are available upon request. For approximate locations see Supplementary Figure 3.

CD74 **primer combinations**	
1F	GTCTAGGGATTGAGCACAGC
1R	ATGACCAGCCTCAGCCATAA
2F	AACATGGGTGATAATCTGGG
2R	TTACACAATGTCCCAGAGCA
3F	TGCTCTGGGACATTGTGTAA
3R	GCACAGTGCCAGGGTACAT
HSP90α **primer combinations**	
1F	GATGCAGATCATTCTCCTGA
1R	CCTACATAGCTATGCAGTTA
2F	TCCCAGATGAACTGGCAAAG
2R	CACCTGCGCACACACAGAG
3F	CTAAGCTGTGATTCACCCGG
3R	GCAGCGAGTGACGTCATCCG
4F	CGGATGACGTCACTCGCTGC
4R	GCGTCAGTGAAGRGAGCAGC
5F	ACTAGAGAGGGTACAGCGGA
5R	GGAAACCGAGCAGCTCCTTG
LCP1 **primer combinations**	
1F	ACTTCAGCCTGTCACTTCCA
1R	CCATCTGCTTCTTCATGCAC
2F	GTTAGATCCATAGTCTTCCC
2R	GCACTCACCAACTTTGCTGA
